# Unintentional weight loss: Clinical characteristics and outcomes in a prospective cohort of 2677 patients

**DOI:** 10.1371/journal.pone.0175125

**Published:** 2017-04-07

**Authors:** Xavier Bosch, Esther Monclús, Ona Escoda, Mar Guerra-García, Pedro Moreno, Neus Guasch, Alfons López-Soto

**Affiliations:** 1 Quick Diagnosis Unit, Adult Day Care Center, Hospital Clínic, University of Barcelona, Barcelona, Spain; 2 Department of Internal Medicine, Hospital Clínic, University of Barcelona, Barcelona, Spain; Providence VA Medical Center, UNITED STATES

## Abstract

**Background:**

Whereas there are numerous studies on unintentional weight loss (UWL), these have been limited by small sample sizes, short or variable follow‐up, and focus on older patients. Although some case series have revealed that malignancies escaping early detection and uncovered subsequently are exceptional, reported follow-ups have been too short or unspecified and necropsies seldom made. Our objective was to examine the etiologies, characteristics, and long-term outcome of UWL in a large cohort of outpatients.

**Methods:**

We prospectively enrolled patients referred to an outpatient diagnosis unit for evaluation of UWL as a dominant or isolated feature of disease. Eligible patients underwent a standard baseline evaluation with laboratory tests and chest X-ray. Patients without identifiable causes 6 months after presentation underwent a systematic follow-up lasting for 60 further months. Subjects aged ≥65 years without initially recognizable causes underwent an oral cavity examination, a videofluoroscopy or swallowing study, and a depression and cognitive assessment.

**Results:**

Overall, 2677 patients (mean age, 64.4 [14.7] years; 51% males) were included. Predominant etiologies were digestive organic disorders (nonmalignant in 17% and malignant in 16%). Psychosocial disorders explained 16% of cases. Oral disorders were second to nonhematologic malignancies as cause of UWL in patients aged ≥65 years. Although 375 (14%) patients were initially diagnosed with unexplained UWL, malignancies were detected in only 19 (5%) within the first 28 months after referral. Diagnosis was established at autopsy in 14 cases.

**Conclusion:**

This investigation provides new information on the relevance of follow-up in the long-term clinical outcome of patients with unexplained UWL and on the role of age on this entity. Although unexplained UWL seldom constitutes a short-term medical alert, malignancies may be undetectable until death. Therefore, these patients should be followed up regularly (eg yearly visits) for longer than reported periods, and autopsies pursued when facing unsolved deaths.

## Introduction

Owing to the broad differential diagnosis and the lack of standardized guidelines or externally validated clinical prediction rules, unintentional weight loss (UWL) constitutes a diagnostic challenge for the clinician. While clinical judgment remains ever essential, the workup needs to be individualized and primarily based on medical history and physical examination findings [1–5]. However, extensive, costly and invasive investigations are sometimes performed so as to not missing an underlying malignancy [1,2].

Although numerous, yet small, studies have investigated UWL, clinical settings, populations, and definition criteria vary considerably [2–9]. The rate of diagnosis after initial workup (the content of which differs by series) ranges from 33 to 60% [2–8]. In general, malignancies, nonmalignant organic disorders, and psychiatric disorders constitute the main etiologic groups of UWL [1,10–14]. Social factors are occasionally considered a separate group or, more commonly, included in the group of psychiatric causes (so-called psychosocial disorders) [10,11,15–17]. Unexplained UWL is comparatively common, accounting for 11–28% of cases [2–9]. Reports have frequently concentrated on elders, in whom UWS is quite prevalent and somewhat behaves as a distinct clinical entity [10,11,15–18]. Because of complex multifactorial and interrelated causes, UWL in older subjects poses a harder diagnostic challenge.

While the largest prospective studies on UWL have mainly admitted patients for workup [2,8,19], the impact of the recent economic regression on health systems has driven some efforts to reduce the huge expenses incurred by hospitalization [20–24]. In Spain, patients needing workup for potentially serious diseases such as UWL have been traditionally admitted in acute hospitals without needing actual therapy [23,25–27]. Whilst inappropriate use of hospital beds surpasses 20% through diverse specialties in Europe, admission for workup is one of the commonest reasons for inappropriate hospitalizations [28,29]. Deficiencies at several levels of the Spanish healthcare system prompted the creation of alternatives to hospitalization, exemplified by hospital-based outpatients’ quick diagnosis units (OQDUs) [23,25,30–33]. Reported advantages over hospitalization are numerous: besides ensuring a time-to-diagnosis similar to the length-of-stay for the same evaluable condition, these clinics decrease emergency department referrals from primary healthcare centers and ease overcrowding, are associated with higher scores of patient reported satisfaction than inpatients, and are significantly cost-saving [31,34–42]. Malignancies constitute the most frequent diagnosis in Spanish OQDUs (18–30%) [35,42].

Since cancer may be the cause of UWL in some patients presenting with few or no accompanying symptoms [2,3,19], a swift workup can be crucial. Because a normally acceptable physical performance argues against hospitalization of subjects referred for UWL investigation, OQDUs appear a suitable setting for their evaluation. Yet their significance for UWL has not been reported to date. The purpose of this study was to investigate the etiologies, characteristics, and long-term clinical outcomes of UWL in a large cohort of patients referred to the OQDU of a tertiary university hospital, the largest thus far reported.

## Materials and methods

### Setting

The OQDU is located in the adult day care center of the Hospital Clínic of Barcelona. With a reference population of about 550,000, most patients are referred to this outpatient unit from 15 primary healthcare centers and the hospital emergency department. Referral criteria, evaluable disorders (which include UWL), characteristics, and functioning of OQDU have been previously reported [36,39]. The general status of evaluable patients must be well-enough to allow them to travel to hospital, then back to home, for visits and investigations.

### Terms and definitions used in this study

#### Unintentional weight loss

Clinical entity whereby the patient does not purposefully set out to lose weight for any reason and when weight loss as a consequence of advanced chronic diseases or their treatments (eg diuretics for heart failure) is excluded [1]. Definition criteria were numerical verification of >5% reduction in usual body weight over the preceding 6–12 months, or, for subjects without numerical documentation, at least two of the following: evidence of change in clothing size, corroboration of the reported weight loss by a relative or friend, and ability to give a numerical estimate of the amount of weight loss [4].

#### Unintentional weight loss of known origin

When a previously reported cause of UWL was identified.

#### Unexplained unintentional weight loss

When a cause of UWL was not identified after an initial clinical evaluation and workup and it remained unknown 6 months after presentation.

#### Intentional weight loss

When the patient did purposefully set out to lose weight through dieting, exercise, self-induced vomiting, use of medications (eg anorexigenic drugs, diuretics, or laxatives), or following bariatric surgery [14].

#### Cachexia syndrome

Clinical entity whereby the patient has lost >5% of weight in <12 months, has an advanced chronic illness (heart failure, respiratory disease [mainly chronic obstructive pulmonary disease and interstitial lung disease], and kidney disease), and has at least three of the following: anorexia, fatigue, decreased muscle strength, low fat-free mass index, and abnormal laboratory tests including increased inflammatory markers, low serum albumin, and anemia [43].

### Study design and population

In this prospective observational study, consecutive patients referred to OQDU for evaluation of UWL were assessed for eligibility. Patients were referred from the emergency department and 15 primary healthcare centers between August 2008 and February 2011. The research ethics committee of the Hospital Clínic, which operated as a central ethics review board for the primary healthcare centers via specific agreements, approved the study. Participants were required to approve a written informed consent for their enrollment and the ethical guidelines of the Declaration of Helsinki were observed. Inclusion criteria were age ≥18 years, definition criteria of UWL [4], and UWL as a dominant or isolated feature of disease. Exclusion criteria were intentional weight loss [14], cachexia syndrome [43], evolving known malignancy, eating disorders, hospital admission within last 3 months, flare-ups of chronic diseases, and poorly controlled chronic pain syndromes. Patients in whom UWL resolved before the first outpatient visit and those lost to follow-up or dead before a diagnosis was made were also excluded.

### Evaluation

#### Baseline evaluation

At first OQDU consultation, a consultant internist asked patients about past and present medical history and performed an exhaustive physical examination. Attention was paid to clinical presentation, accompanying symptoms, estimated amount of weight loss (percentage and Kg over the preceding 6–12 months), use of prescribed, over-the-counter and illicit drugs, comorbidities (evaluated with the age-adjusted Charlson index [44,45]), psychosocial issues, and, especially in patients aged ≥65 years, presence of dental pain, dry mouth and difficulty swallowing (herein referred to as oral problems or disorders), cognitive impairment/dementia, and disability/immobility. When there were doubts about the presence of cognitive impairment, a relative/caregiver was asked to provide information. Although initial tests were carried out as appropriate according to clinical evaluation and patient age, minimal investigations included chest X-ray and standard laboratory tests. These included C-reactive protein, erythrosedimentation rate, hemogram (total leukocytes, manual white blood cell count, hemoglobin, hematocrit, reticulocytes, red blood cell indices, and platelet count), and serum levels of ferritin, glucose, cholesterol, aminotransferases, alkaline phosphatase, γ-glutamyl transpeptidase, total bilirubin, lactate dehydrogenase, total proteins, albumin, creatinine, and electrolytes (sodium, potassium, and calcium). Protein electrophoresis, estimated glomerular filtration rate, urinalysis, and fecal occult blood were also ordered.

#### Further investigations

Other examinations were ordered according to the results of baseline evaluation and assessment of patient outcome during successive visits (eg additional laboratory tests, imaging studies such as abdominal ultrasound, computed tomography and FDG-PET scanning, endoscopies, and biopsy/cytology studies). Patients aged ≥65 years with unexplained UWL after the first evaluation underwent an examination of the oral cavity. Potential depression and cognitive problems were assessed with the geriatric depression scale (short form) [46] and the Mini-Cognitive Assessment Instrument (Mini-Cog) [47], respectively. Uncertain difficulty swallowing/dysphagia was evaluated with videofluoroscopy, or, if unfeasible, a swallowing study [48]. The latter is an adaptation of a validated procedure in which subjective bedside assessment of swallowing is combined with concurrent monitoring of oxygen saturation [48,49].

### Causes of unintentional weight loss

The individual causes of UWL were coded *post hoc* according to the International Statistical Classification of Diseases and Related Health Problems, Tenth Revision (ICD-10 Version: 2015) [50]. Specifically, a list was first created that included all individual diagnosis reported to cause UWL. Each listed disorder was then searched in the ICD-10 list and codified accordingly. When any disorder was not termed as such in the ICD-10 list, the equivalent disorder and the corresponding code were used. For instance, diagnosis ‘oral, swallowing, and dental problems’ does not exist as such in the ICD-10 list. However, codes K08.9, K11.7, R13, and R43.8 in the list correspond to ‘disorder of teeth and supporting structures, unspecified’, ‘disturbances of salivary secretion’, ‘dysphagia (*Incl*.: *difficulty in swallowing*)’, and ‘other and unspecified disturbances of smell and taste’, respectively. Furthermore, to ensure accuracy in the validation of the codification process, three coauthors (EM, OE, and PM) independently performed this process for all the individual diagnoses. Any discrepancy was eventually resolved by consensus with the senior author.

In line with earlier reports [1–3,8,9], albeit with some variation, three main etiologic groups were established: malignancies, nonmalignant organic disorders, and psychosocial disorders.

### Follow-up

All patients were initially followed-up for a minimum of 1 to a maximum of 18 months or death.

#### Unexplained unintentional weight loss

Patients with unexplained UWL were followed-up for localizing symptoms or signs suggesting a diagnosis, weight changes, and survival. Patients were firstly reevaluated through successive visits at 1, 3, and 6 months after presentation. When the cause remained unknown after the initial 6 months’ period, reevaluation continued by further visits at 9, 12, and 18 months or, if the patient missed the appointment, by phone calls to them (or their relatives) and the physicians at primary healthcare centers in charge of them. If the etiology still remained unknown 18 months after presentation, the primary healthcare centers’ physicians and the patients (or their relatives) were interviewed by the OQDU physicians once every 3 months to a maximum of 4 additional years or death. Patients’ electronic health records—recorded in the integrated computerized system of the hospital and the primary healthcare centers—were also accessed and examined to obtain follow-up clinical data.

#### Unintentional weight loss of known origin

Patients were followed-up after diagnosis for weight changes and survival in each etiologic group. Electronic medical records were also accessed to obtain follow-up information. If it was insufficient, the patients (or their relatives) or the physicians in charge of them were contacted.

### Database

Besides the aforementioned data, referral sources, demographic and epidemiological data, socioeconomic status (measured by education and income), smoking habit, alcohol consumption, type and number of investigations performed, time-to-diagnosis (from first OQDU visit to diagnosis), and hospitalizations were prospectively tabulated in a database.

### Statistical analysis

The chi-square test or the Fisher’s exact test, as appropriate, was used to compare categorical data, which are expressed as absolute frequencies (%). The *t*-test was used to compare continuous variables with a normal distribution, which are expressed as means with standard deviations (SD). When appropriate, the nonparametric Mann-Whitney *U* test was used to compare continuous variables with skewed distributions. Since it was a prospective study where all data were meticulously collected and registered, the amount of missing data was expected to be irrelevant. However, any type of missing items was included in the study analysis. Statistical significance was established at .05. Analyses were done with SPSS software (version 21.0) (SPSS, Chicago, USA).

## Results

### General characteristics of study population

**Fig 1:**
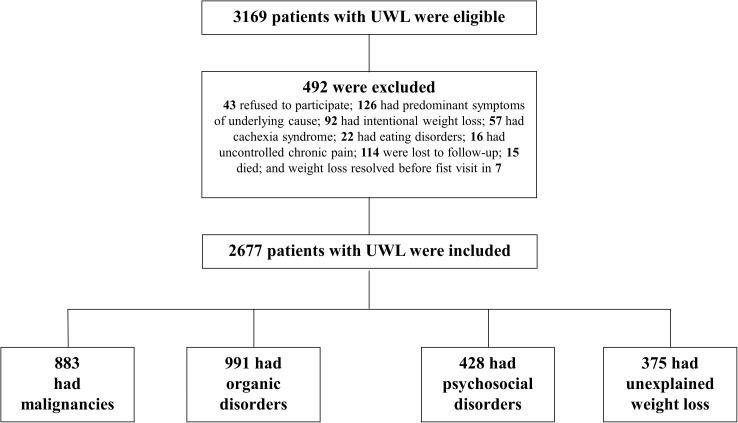
Flowchart of enrollment of patients with unintentional weight loss.

Among 3169 initially eligible patients, 574 were excluded, leaving 2677 for the final analysis (Fig 1). Fifty-one percent were males and mean age was 64.4 (14.7) years. The estimated weight loss was 11.9 (6.7) percent or 8.3 (4.5) Kg. Referral sources, socioeconomic status, smoking and drinking status, and mean time-to-diagnosis are shown in Table 1. There were minimal missing data on the categorical variables educational level and monthly income (0.001 and <1%, respectively) and complete data on other variables. The relative percentages of the items of these categorical variables were calculated according to the total number of patients without missing data (see footnote of Table 1).

**Table 1:** General characteristics of study patients.

Characteristic	Patients
	(N = 2677)
**Sources of referral, n (%)**	
Emergency department	1660 (62)
Primary healthcare centers	1017 (38)
Age (years), mean (SD)	64.4 (14.7)
**Sex, n (%)**	
Females	1306 (49)
Males	1371 (51)
**Socioeconomic status**	
*Educational level*, *n (%)*	
No schooling	252 (9)
Primary or lower secondary	1043 (39)
Upper secondary or professional training	949 (35)
University	430 (16)
*Monthly income (Euros)*, *n (%)* ^*^	
≤900	274 (10)
901–1200	809 (30)
1201–1800	984 (37)
>1800	590 (22)
Active smoker, n (%)	773 (29)
Active drinker, n (%)	570 (21)
Estimated weight loss (% over 6–12 months), mean (SD)	11.9 (6.7)
Estimated weight loss (Kg over 6–12 months), mean (SD)	8.3 (4.5)
**Unintentional weight loss of known origin, n (%)**	2302 (86)
Malignant disorders	883 (33)
Nonmalignant organic disorders	991 (37)
Psychosocial disorders	428 (16)
**Initially unexplained unintentional weight loss, n (%)** ^**†**^	375 (14)
Time-to-diagnosis (days), mean (SD) ^‡^	14.0 (3.6)

^*^ Self-reported household income, including all income components received by any household member, after tax subtraction.

^†^ Patients without an identifiable cause of unintentional weight loss 6 months after presentation.

^‡^ For malignant disorders, nonmalignant organic disorders, and psychosocial disorders.

Missing data: variables ‘no schooling’ (n = 3) and monthly incomes of’ ‘901–1200’ (n = 8) and of ‘1201–1800’ (n = 12).

### Causes of unintentional weight loss

Although the cause of UWL was unknown in 582 patients after the first outpatient visit, evaluation within 6 months after presentation disclosed an etiology in 207 including malignancies in 27, digestive disorders in 13, polyarteritis nodosa in 3, UWL as a side effect of drugs (herein referred to as drug-induced UWL) in 24, and psychosocial disorders in 140. Thus, 375 patients remained without diagnosis and were categorized with unexplained UWL (14% of patients) and the rest (86%) were categorized with UWL of known origin. Specific causal disorders and ICD-10 codes matched in 96% of cases after an independent assessment by three investigators. Agreement was reached in all cases after discussion with the leading investigator. For the purpose of the analyses, 4 main groups of UWL were established: nonmalignant organic disorders (n = 991 [37%]), malignancies (n = 883 [33%]), psychosocial disorders (n = 428 [16%]), and unexplained UWL (n = 375 [14%]) (Fig 1 and Tables 1 and 2).

**Table 2:** Specific causes of unintentional weight loss.*

Cause	Patients ^†^
	(N = 2677)
**Malignant disorders**	883 (33)
Esophagus, stomach, colon, rectum, pancreas, liver cell carcinoma, and intrahepatic bile duct carcinoma *[C15*, *C16*, *C18*, *C20*, *C25*, *C22*.*0*, *C22*.*1]*	415 (47)
Breast *[C50]*	27 (3)
Cervix uteri, endometrium, and ovary *[C53*, *C54*.*1*, *C56]*	35 (4)
Prostate *[C61]*	53 (6)
Kidney, renal pelvis, ureter, and bladder *[C64*, *C65*, *C66*, *C67]*	88 (10)
Bronchus and lung *[C34]*	150 (17)
Hodgkin lymphoma and non-Hodgkin lymphoma, unspecified *[C81*, *C85*.*9]*	97 (11)
Malignant neoplasm, primary site unknown, so stated *[C80*.*0]*	18 (2)
**Nonmalignant organic disorders**	991 (37)
*Digestive disorders*	450 (45)
Gastric and duodenal ulcers *[K25*, *K26]*	81 (18)
Oral, swallowing, and dental problems *[K08*.*9*, *K11*.*7*, *R13*, *R43*.*8]*	141 (31)
Crohn disease [regional enteritis] and ulcerative colitis *[K50*, *K51]*	44 (10)
Indeterminate colitis and other specified noninfective gastroenteritis and colitis *[K52*.*3*, *K52*.*8]*	97 (22)
Chronic vascular disorders of intestine *[K55*.*1]*	24 (5)
Celiac disease, pancreatic steatorrhea, and intestinal malabsorption, unspecified *[K90*.*0*, *K90*.*3*, *K90*.*9]*	63 (14)
*Endocrine disorders*	139 (14)
Thyrotoxicosis [hyperthyroidism] *[E05]*	64 (46)
Subacute thyroiditis *[E06*.*1]*	11 (8)
Type 1 and type 2 diabetes mellitus *[E10*, *E11]*	54 (39)
Primary hyperparathyroidism *[E21*.*0]*	10 (7)
*Infectious diseases*	119 (12)
Primary respiratory tuberculosis, tuberculous pleurisy and tuberculosis of intrathoracic lymph nodes, without mention of bacteriological or histological confirmation, and tuberculous peripheral lymphadenopathy *[A16*.*7*, *A16*.*5*, *A16*.*3*, *A18*.*2]*	46 (39)
Human immunodeficiency virus [HIV] disease resulting in infectious and parasitic diseases, malignant neoplasms, and wasting syndrome *[B20*, *B21*, *B22*.*2]*	26 (22)
Other gastroenteritis and colitis of infectious and unspecified origin *[A09]*	45 (38)
Acute and subacute infective endocarditis *[I33*.*0]*	2 (2)
*Nervous system diseases*	80 (8)
Stroke, not specified as hemorrhage or infarction *[I64]*	46 (58)
Parkinson disease *[G20]*	30 (38)
Motor neuron disease *[G12*.*2]*	4 (5)
*Respiratory diseases*	20 (2)
Interstitial pulmonary disease, unspecified, and other interstitial pulmonary diseases with fibrosis *[J84*.*9*, *J84*.*1]*	4 (20)
Pleural effusion, not elsewhere classified, and pyothorax without fistula *[J90*, *J86*.*9]*	14 (70)
Abscess of lung without pneumonia *[J85*.*2]*	2 (10)
*Systemic autoimmune diseases*	89 (9)
Polymyalgia rheumatica and giant cell arteritis with polymyalgia rheumatica *[M35*.*3*, *M31*.*5]*	56 (63)
Polyarteritis nodosa *[M30*.*0]*	9 (10)
Systemic lupus erythematosus with organ or system involvement and rheumatoid arthritis, unspecified *[M32*.*1*^*†*^, *M06*.*9]*	24 (27)
*Kidney and ureteral diseases*	40 (4)
Rapidly progressive nephritic syndrome, chronic nephritic syndrome, and nephrotic syndrome *[N01*, *N03*, *N04]*	12 (30)
Nonobstructive reflux-associated chronic pyelonephritis, chronic obstructive pyelonephritis, and other andunspecified hydronephrosis *[N11*.*0*, *N11*.*1*, *N13*.*3]*	28 (70)
*Unspecified adverse effect of drug or medicament [T88*.*7]* ^‡^	54 (5)
**Psychosocial disorders**	428 (16)
Depressive episode *[F32]*	180 (42)
Somatoform disorders, other anxiety disorders, and obsessive-compulsive disorder *[F45*, *F41*, *F42]*	122 (29)
Bipolar affective disorder and schizophrenia *[F31*, *F20]*	21 (5)
Mental and behavioral disorders due to use of alcohol, opioids, cocaine, and multiple drug use and use of other psychoactive substances *[F10*, *F11*, *F14*, *F19]*	4 (1)
Dementia in Alzheimer disease, unspecified, vascular dementia, unspecified, and unspecified dementia *[F00*.9^*^, *F01*.*9*, *F03]*	47 (11)
Problems related to housing and economic circumstances *[Z59]*	30 (7)
Problems related to social environment including social exclusion and rejection, and neglect and abandonment *[Z60*, *Y06]*	18 (4)
Immobility and limitation of activities due to disability *[R26*.*3*, *Z73*.*6]*	6 (1)
**Initially unexplained unintentional weight loss**	375 (14)

^*^ Causes are categorized according to the International Statistical Classification of Diseases and Related Health Problems 10^th^ Revision (ICD-10 Version: 2015) [50].

^**†**^ Meaning of the percentages (numbers in parentheses) listed in the table: percentages in main groups (malignant, nonmalignant organic, and psychosocial disorders) are relative to the entire sample size; percentages in individual malignant disorders and in individual psychosocial disorders are relative to the total number of malignant disorders and the total number of psychosocial disorders, respectively; percentages in the main subgroups of nonmalignant organic disorders (ie digestive, endocrine, infectious nervous system, respiratory, systemic autoimmune, and kidney and ureteral disorders as well as adverse effect of drug) are relative to the total number of nonmalignant organic disorders; and percentages in the individual conditions of each subgroup n of nonmalignant organic disorders are relative to the total number of each subgroup.

^‡^ Formerly reported prescription, over-the-counter, and illicit drugs accounting for unintentional weight loss.

#### Malignancies

Manifestations leading to a diagnosis within the first 6 months in the 27 abovementioned cancer patients included, among others, abdominal pain, fever, and peripheral lymphadenopathy. Out of 27, 11 had pancreatic cancer and 9 had lymphoma. Overall, digestive cancers (n = 415 [47% of malignancies]) prevailed over the rest of cancers. Main specific cancers were pancreatic (19% of malignancies), lung (17%), lymphoma (11%), and kidney, ureteral, and bladder cancers (10% together) (Table 2).

#### Nonmalignant organic disorders

Most common disorders were digestive (n = 450 [45% of nonmalignant organic disorders]), with oral disorders making up 31% of digestive causes. Endocrine, infectious, nervous system and systemic autoimmune diseases, and drug-induced UWL were also relatively frequent (Table 2).

#### Psychiatric disorders

Psychiatric causes were diagnosed in 374 (14%) patients, most notably depression (7% of UWL cases). Social factors accounted for 2% of UWL cases, mostly comprising problems related to housing and economic circumstances (Table 2).

#### Characteristics according to patient groups

Patients with malignancies were older, more often males and active smokers, and had a greater weight loss than patients from the other groups. While patients with nonmalignant organic disorders were younger than other patients, those with psychosocial disorders were more often females and had a lower socioeconomic status and a smaller amount of weight loss than others. Patents with malignancies and nonmalignant organic disorders were more likely to have accompanying symptoms and abnormalities on physical examination, laboratory tests and chest X-ray and less often had a normal baseline evaluation than patients with psychosocial disorders and those with unexplained UWL. Time-to-diagnosis in the former two groups was statistically longer that in patients with psychosocial disorders. However, this had a minimal, if any, clinical relevance since mean differences were only 2.3 and 3.1 days, respectively (Table 3).

**Table 3:** Characteristics of patient groups.

Characteristic	MDs *a*	NMDs *b*	PSDs *c*	UE *d*	*P* value					
	(n = 883)	(n = 991)	(n = 428)	(n = 375)	*a* vs. *b*	*a* vs. *c*	*a* vs. *d*	*b* vs. *c*	*b* vs. *d*	*c* vs. *d*
Age (years), mean (SD)	69.1 (12.2)	60.2 (12.8)	62.7 (11.0)	65.1 (10.4)	< .001	< .001	< .001	< .001	< .001	< .001
**Sex, n (%)**					< .001	< .001	< .001	.101	.044	< .001
Females	362 (41)	525 (53)	235 (55)	184 (49)						
Males	521 (59)	466 (47)	193 (45)	191 (51)						
**SE status**										
*Education*, *n (%)*					.048	< .001	.123	< .001	.102	< .001
No schooling	86 (10)	68 (7)	64 (15)	34 (9)						
Primary/lower secondary	353 (40)	347 (35)	205 (48)	138 (37)						
Upper secondary/PT	309 (35)	377 (38)	124 (29)	139 (37)						
University	133 (15)	198 (20)	35 (8)	64 (17)						
*Monthly income (Euros)*, *n (%)*					< .001	.112	< .001	< .001	.127	< .001
≤900	115 (13)	69 (7)	60 (14)	30 (8)						
901–1200	296 (34)	255 (26)	157 (37)	101 (27)						
1201–1800	313 (36)	382 (39)	141 (33)	148 (40)						
>1800	150 (17)	277 (28)	69 (16)	94 (25)						
Active smoker, n (%)	291 (33)	277 (28)	111 (26)	94 (25)	< .001	< .001	< .001	.113	.100	.234
Active drinker, n (%)	203 (23)	198 (20)	94 (22)	75 (20)	.060	.162	.075	.102	.301	.129
Estimated WL (%), mean (SD)	14.9 (5.9)	11.5 (6.5)	10.3 (4.6)	10.8 (4.3)	< .001	< .001	< .001	.047	.080	.200
Estimated WL (Kg), mean (SD)	10.4 (4.0)	8.1 (4.2)	7.2 (3.1)	7.6 (2.9)	< .001	< .001	< .001	.039	.095	.217
**Baseline evaluation, n (%)**										
Accompanying symptoms	547 (62)	763 (77)	13 (3)	30 (8)	< .001	< .001	< .001	< .001	< .001	.042
PE abnormalities	424 (48)	367 (37)	5 (1)	8 (2)	< .001	< .001	< .001	< .001	< .001	.248
Lab. abnormalities	865 (98)	651 (66)	42 (10)	68 (18)	< .001	< .001	< .001	< .001	< .001	< .001
Chest X-ray abnormalities	142 (16)	66 (7)	3 (1)	4 (1)	< .001	< .001	< .001	< .001	< .001	.315
Normal baseline evaluation	4 (0)	55 (6)	364 (85)	272 (73)	< .001	< .001	< .001	< .001	< .001	< .001
Time-to-diagnosis (days), mean (SD) ^*^	14.6 (3.1)	15.4 (3.3)	12.3 (2.5)	na.	.057	< .001	na.	< .001	na.	na.

MDs, malignant disorders; NMDs, nonmalignant organic disorders; PSDs, psychosocial disorders; UE, initially unexplained unintentional weight loss; SE, socioeconomic; PT, professional training; WL, weight loss; PE, physical examination; Lab., laboratory; na., not applicable.

^*^ Initially unexplained unintentional weight loss is excluded.

Missing data: variables ‘no schooling’ (n = 2 in MDs and 1 in NMDs) and monthly incomes of’ ‘901–1200’

(n = 4 in MDs, 3 in NMDs, and 1 in PSDs) and of ‘1201–1800’ (n = 5 in MDs, 5 in NMDs, and 2 in UE).

### Characteristics according to age

When comparing patients aged <65 years (n = 1258) to those aged ≥65 years (n = 1419), several significant differences were observed (Table 4). Older subjects were more likely than their younger counterparts to be males and to have more comorbidities and more weight loss but were less often diagnosed with UWL of known origin (83 vs. 89%). Malignancies in general, and digestive and other nonhematologic malignancies in particular, were more prevalent in older patients, whereas hematologic malignancies prevailed in younger subjects. Oral disorders, stroke, Parkinson disease, dementia, polymyalgia rheumatica with/without giant-cell arteritis, and drug-induced UWL were more common in older patients. In contrast, endocrine disorders, tuberculosis, HIV disease, and depressive, anxiety, and obsessive-compulsive disorders prevailed in younger patients (Table 4). In addition to educational level and monthly income, there was a minimal rate of missing data on the variable age-adjusted Charlson comorbidity index (<1% in both patients aged <65 and ≥65 years) (see footnote of Table 4).

**Table 4:** Characteristics according to age.*^†^

Characteristic	<65 years	≥65 years	*P* value
	(n = 1258)	(n = 1419)	
**Sex, n (%)**			.046
Females	634 (50)	670 (47)	
Males	624 (50)	749 (53)	
**Socioeconomic status**			
*Educational level*, *n (%)*			.106
No schooling	125 (10)	127 (9)	
Primary or lower secondary	504 (40)	539 (38)	
Upper secondary or professional training	424 (34)	525 (37)	
University	203 (16)	227 (16)	
*Monthly household income (Euros)*, *n (%)*			.067
≤900	118 (9)	156 (11)	
901–1200	331 (26)	478 (34)	
1201–1800	466 (37)	518 (37)	
>1800	335 (27)	255 (18)	
Current smoker, n (%)	347 (28)	426 (30)	.079
Current drinker, n (%)	262 (21)	308 (22)	.124
Age-adjusted Charlson comorbidity index (score), mean (SD)	2.4 (1.2)	4.9 (1.8)	< .001
Estimated weight loss (% over 6–12 months), mean (SD)	10.7 (6.6)	13.1 (7.2)	< .001
Estimated weight loss (Kg over 6–12 months), mean (SD)	7.5 (4.4)	9.1 (5.1)	< .001
**Unintentional weight loss of known origin, n (%)**	1121 (89)	1181 (83)	< .001
***Malignant disorders***	390 (31)	493 (35)	< .001
Digestive malignancies *[C15*, *C16*, *C18*, *C20*, *C25*, *C22*.*0*, *C22*.*1]*	170 (14 [44])	245 (17 [50])	.032
Other nonhematologic malignancies *[C50*, *C53*, *C54*.*1*, *C56*, *C61*, *C64*, *C65*, *C66*, *C67*, *C34*, *C80*.*0]*	144 (11 [37])	227 (16 [46])	< .001
Hematologic malignancies *[C81*, *C85*.*9]*	76 (6 [19])	21 (1 [4])	< .001
***Nonmalignant organic disorders***	479 (38)	512 (36)	.072
*Digestive disorders*	191 (15)	259 (18)	.040
Oral/swallowing/dental problems *[R13*, *K11*.*7*, *K08*.*9*, *R43*.*8]* ^‡^	20 (2 [10])	121 (9 [47])	< .001
Noninfective colitis/gastroenteritis *[K52*.*3*, *K52*.*8]*	58 (5 [30])	39 (3 [15])	.090
Chronic vascular intestinal disorders *[K55*.*1]*	2 (0 [1])	22 (2 [8])	.039
Other digestive disorders *[K25*, *K26*, *K50*, *K51*, *K90*.*0*, *K90*.*3*, *K90*.*9]*	111 (9 [58])	77 (5 [30])	< .001
*Endocrine disorders [E05*, *E06*.*1*, *E10*, *E11*, *E21*.*0]*	106 (8)	33 (2)	< .001
*Infectious diseases*	99 (8)	20 (1)	< .001
Tuberculosis *[A16*.*7*, *A16*.*5*, *A18*.*2*, *A16*.*3]*	39 (3 [39])	7 (0 [35])	.024
Human immunodeficiency virus [HIV] disease *[B20*, *B21*, *B22*.*2]*	24 (2 [24])	2 (0 [10])	.028
Gastroenteritis/colitis *[A09]*	35 (3 [35])	10 (1 [50])	.093
Infective endocarditis *[I33*.*0]*	1 (0 [1])	1 (0 [5])	.203
*Nervous system diseases*	18 (1)	62 (4)	.037
Stroke and Parkinson disease *[I64*, *G20]*	15 (1 [83])	61 (4 [98])	.033
Motor neuron *disease [G12*.*2]*	3 (0 [17])	1 (0 [2])	.199
*Respiratory diseases [J84*.*1*, *J84*.*9*, *J90*, *J86*.*9*, *J85*.*2]*	10 (1)	10 (1)	.287
*Systemic autoimmune diseases*	26 (2)	63 (4)	.072
Polymyalgia rheumatica/giant cell arteritis *[M35*.*3*, *M31*.*5]*	4 (0 [15])	52 (4 [83])	< .001
Other autoimmune diseases *[M30*.*0*, *M32*.*1*^**†**^, *M06*.*9]*	22 (2 [85])	11 (1 [17])	.136
*Kidney and ureteral diseases [N01*, *N03*, *N04*, *N11*.*0*, *N11*.*1*, *N13*.*3]*	26 (2)	14 (1)	.140
*Adverse effect of drugs [T88*.*7]*	3 (0)	51 (4)	< .001
***Psychosocial disorders***	252 (20)	176 (12)	< .001
Depression/anxiety disorders/obsessive-compulsive disorder *[F32*, *F45*, *F41*, *F42]*	193 (15 [77])	109 (8 [62])	< .001
Dementia *[F00*.9^*^, *F01*.*9*, *F03]*	5 (0 [2])	42 (3 [24])	.019
Socioeconomic factors and immobility/disability problems *[Z59*, *Z60*, *R26*.*3*, *Z73*.*6]*	32 (3 [13])	22 (2 [13])	.140
Other psychiatric disorders *[F31*, *F20*, *F10*, *F11*, *F14*, *F19]*	22 (2 [9])	3 (0 [2])	.084
**Initially unexplained unintentional weight loss, n (%)**	137 (11)	238 (17)	< .001
Time-to-diagnosis (days), mean (SD)	13.5 (3.4)	14.6 (3.5)	.046
*Follow-up*			
Follow-up time (months), mean (SD)	14.9 (7.8)	13.9 (7.0)	.058
Deaths, n (%)	282 (22)	440 (31)	< .001

* ^*^ Causes are categorized according to the International Statistical Classification of Diseases and Related Health Problems 10^th^ Revision (ICD-10 Version: 2015) [50]. Codes for specific disorders are detailed in Table 2.

^†^ Relative percentages are shown in square brackets.

^‡^ R13: dysphagia and difficulty in swallowing, K11.7: disturbances of salivary secretion, K08.9: disorder of teeth and supporting structures, unspecified, R43.8: other and unspecified disturbances of smell and taste [50].

Missing data: variables ‘no schooling’ (n = 2 in patients <65 years and n = 1 in patients ≥65 years), monthly incomes of ‘901–1200’ (n = 3 in patients <65 years and n = 5 in patients ≥65 years) and of ‘1201–1800’ (n = 5 in patients <65 years and n = 7 in patients ≥65 years), and ‘age-adjusted Charlson comorbidity index’ (n = 8 in patients <65 years and n = 11 in patients ≥65 years).

### Clinical outcome of patient groups

Patients were initially followed-up for a mean of 14.5 (7.6) months (range, 1–18 months). Table 5 displays the mean follow-up times and mortality rates in each group. A total of 712 (27% of study population) patients died during the initial follow-up. Cancer patients had the highest mortality rate (69 vs. 5–6% in other groups). Survivors in each group were categorized according to weight changes (decreased, increased, or stable). Decreasing weight was substantially more frequent in survivors with malignancies (66%) than in the rest and it was also more common in survivors with nonmalignant organic disorders (10%) than in unexplained UWL (6%) and psychosocial disorders (3%). Increasing weight was more common in patients with psychosocial disorders (55%) than in nonmalignant organic disorders (39%), unexplained UWL (35%), and malignancies (12%). Stable weight was more often in survivors with unexplained UWL (59%) than in nonmalignant organic disorders (51%), psychosocial disorders (42%), and malignancies (22%) (Table 5).

**Table 5:** Eighteen months’ outcomes of patient groups.

Outcome	MDs *a*	NMDs *b*	PSDs *c*	UE *d*	*P* value					
	(n = 883)	(n = 991)	(n = 428)	(n = 375)	*a* vs. *b*	*a* vs. *c*	*a* vs. *d*	*b* vs. *c*	*b* vs. *d*	*c* vs. *d*
Follow-up time (months), mean (SD)	9.7 (5.3)	16.2 (6.2)	17.3 (7.4)	16.8 (6.9)	< .001	< .001	< .001	.077	.138	.216
Deaths, n (%)	612 (69)	61 (6)	21 (5)	18 (5)	< .001	< .001	< .001	.172	.146	.274
Survivors with increased weight, n (%)	29/238 (12)	345/885(39)	205/373(55)	120/347 (35)	< .001	< .001	< .001	< .001	.042	< .001
Survivors with decreased weight, n (%)	157/238 (66)	89/885(10)	11/373(3)	22/347(6)	< .001	< .001	< .001	< .001	.031	.105
Survivors with stable weight, n (%)	52/238(22)	451/885(51)	157/373(42)	205/347(59)	< .001	< .001	< .001	< .001	< .001	< .001
Lost to follow-up, n (%)	33 (4)	45 (5)	34 (8)	10 (3)						

MDs, malignant disorders; NMDs, nonmalignant organic disorders; PSDs, psychosocial disorders; UE, initially unexplained unintentional weight loss.

#### Extended follow-up in patients with unexplained unintentional weight loss

Patients with unexplained UWL were followed up for an initial period of 16.8 (6.9) months and an additional period of 33.7 (9.4) months. The cumulative mean follow-up period (from initial presentation) of the 375 patients with unexplained UWL was 47.5 (10.8) months (range, 1–65 months).

During the initial period (1–18 months), 18 patients died—16 shortly after hospitalization—and 10 were lost to follow-up. Thirteen of 18 underwent autopsy. Specifically, autopsies were performed in 10 patients with ongoing weight loss and in 3 in whom weight loss had stabilized. All 13 autopsied patients died between 7 and 15 months after presentation. Postmortem examination revealed malignancies in 8 patients with slowly progressive, ongoing weight loss including non-Hodgkin lymphoma in 4, metastatic melanoma in 3, and renal-cell carcinoma in 1 along with massive pulmonary thromboembolism in 5 of them. There was no evidence of malignancy in the remaining 5 autopsied patients. In addition, a likely underlying cause was recognized in 4 additional patients with persistent weight loss including non-Hodgkin lymphoma in 2, metastatic melanoma in 1, and disseminated germ cell tumor in 1. All 4 patients were diagnosed between 6 and 13 months after presentation. During the additional follow-up period (18–66 months), 54 patients died—43 shortly after hospitalization—and 27 were lost to follow-up. Twenty-four of 54 underwent autopsy between 18 and 28 months after presentation. Necropsies were performed in 8 patients who had continued decreasing weight during the initial period and in whom weight loss continued during the additional period and in 16 patients with stabilized weight in whom weight loss relapsed during the additional period. Examination disclosed malignancies in 6 patients with slow, gradual weight loss including non-Hodgkin lymphoma in 3, metastatic melanoma in 1, renal-cell carcinoma in 1, and carcinoma of unknown primary in 1. The remaining 18 patients did not have evidence of any malignancy at autopsy. Lastly, 1 patient was diagnosed with Hodgkin lymphoma with exclusive bone marrow involvement 19 months after presentation, at time of weight loss relapse following a 2 months’ period of stabilization. Table 6 shows the characteristics of the 19 patients diagnosed with malignancy during follow-up.

**Table 6:** Characteristics of 19 patients diagnosed with cancer during follow-up.

Age (yrs)	Sex	Earlier man. ^*^	Earlier lab. abn.	Earlier Dx tests	Key man. at FU	Decisive Dx proc.	Dx time (mo) ^†^	Cancer type
72	M	None	None	Normal CT & FDG-PET	None	Necropsy	7	NHL
45	M	None	None	Normal CT **×** 2	DVT	RP mass Bx	13	GCT
68	F	None	Mild anemia Thrombocytosis	Normal CT **×** 2 Normal BM asp.	None	Necropsy	9	RCC
70	M	Sweating	Mildly elevated LDH	Normal CT **×** 3 & FDG-PET **×** 2	None	Necropsy	18	NHL
66	F	None	None	Normal CT & FDG-PET	None	Necropsy	9	MM
68	F	Low-grade fever & sweating	Mildly elevated LDH & ESR	Normal CT **×** 2	Fever	BM Bx	6	NHL
63	M	None	None	Normal CT & FDG-PET **×** 2	None	Necropsy	8	MM
71	M	None	None	Normal CT **×** 2 & FDG-PET	None	Necropsy	10	NHL
69	F	None	None	Normal CT **×** 2	Axillary LDP	LDP Bx	8	NHL
74	M	None	None	Normal CT **×** 3 & FDG-PET **×** 3	None	Necropsy	21	RCC
64	F	None	None	Normal CT **×** 2 & FDG-PET	None	Necropsy	7	MM
65	F	None	None	Normal CT **×** 3 & FDG-PET **×** 2	None	Necropsy	19	MM
56	M	None	None	Normal CT **×** 4 & FDG-PET **×** 4	None	Necropsy	28	CUP
69	F	Splenomegaly	None	Normal CT **×** 3 & FDG-PET **×** 3 Normal BM Bx	None	Necropsy	15	NHL
66	M	None	None	Normal CT & FDG-PET	Lumbar pain	Bone Bx	7	MM
67	M	None	Leukocytosis Elevated AST, ALT & GGT	Normal CT **×** 3 & FDG-PET **×** 2 Normal liver Bx	None	Necropsy	18	NHL
73	F	None	None	Normal CT **×** 3 & FDG-PET **×** 3	Fever	Necropsy	20	NHL
60	F	Low-grade fever	None	Normal CT **×** 4 & FDG-PET **×** 2	High fever	BM Bx	19	HL
70	M	None	None	Normal CT **×** 2 & FDG-PET	None	Necropsy	11	NHL

M, male; F, female; man., manifestations; lab., laboratory; abn., abnormalities; Dx, diagnostic; FU, follow-up; CT, computed tomography; FDG-PET, fludeoxyglucose-positron emission tomography; NHL, non-Hodgkin lymphoma; DVT, deep vein thrombosis; RP, retroperitoneal; Bx, biopsy; GCT, germ cell tumor; BM, bone marrow; asp., aspiration; RCC, renal-cell cancer; LDH, lactate dehydrogenase; MM, metastatic melanoma; ESR, erythrocyte sedimentation rate; LDP, lymphadenopathy; CUP, carcinoma of unknown primary; AST, aspartate transaminase; ALT, alanine transaminase; GGT, gamma glutamyl transpeptidase; HL, Hodgkin lymphoma.

^*^ Other than weight loss.

^†^ Time elapsed between initial presentation and definitive diagnosis.

## Discussion

We report here the largest prospective study of patients with UWL. While the main etiologies of UWL agreed in general with previous reports, a salient finding of the study was the long follow-up of patients with a diagnosis of unexplained UWL. During this period, the longest reported up to now, several malignancies were detected in some living and, remarkably, deceased patients at postmortem studies. Additionally, relevant differences were observed between patients aged ≥65 years and those aged <65 years. The sample sizes of the two groups were large enough to establish solid comparisons, which may help further understand the complex relationship between age and UWL.

With nearly 40% of UWL causes, nonmalignant organic disorders were the most frequent etiologic group. Consistent with preceding reports [2–4,6,8,9], digestive disorders largely predominated in this group (45%). Of note, oral problems accounted for more digestive causes of UWL than any other digestive condition—a factor linked to patient age (see below). Malignancies were identified in one third of UWL patients and, also in line with reported evidence, digestive malignancies prevailed (47%) [2,3,9,19], with pancreatic cancer comprising almost 20% of all malignancies. As expected [3,9], cancer patients were older, more often male, and had more pronounced weight loss than other patients. While psychosocial disorders (principally depression) explained UWL in 16% of patients, an initial cause was not identified in 14%.

Although UWL has been extensively investigated in elders [1,10,11,15–18], studies have not explicitly analyzed the differences between older and younger patients. Reports indicate that 15–20% of adults aged ≥65 years have UWL [10,11] and that this prevalence is higher in community dwelling elders (27%) and nursing home residents (50–60%) [51]. Similar to the etiologies irrespective of age, malignancies, nonmalignant organic disorders, and psychosocial disorders were the most common causes of UWL in older subjects in the current study. Expectedly, older patients had a higher occurrence of malignancies and specific nonmalignant organic disorders than younger patients, including nervous system diseases, polymyalgia rheumatica with/without giant-cell arteritis, drug-induced UWL, and, predominantly, oral problems. The latter were only surpassed by nonhematologic malignancies as a cause of UWL. While oral disorders made up 47% of digestive causes or 9% of all UWL causes in patients aged ≥65 years, the figures in younger patients were 10 and 2%, respectively.

Several physiological and psychosocial factors may cause or contribute to UWL in elders. Besides the decrease in lean body mass and bone mass with ageing, premature satiation due to distorted gastric signals [52] and reduced senses of smell and taste [1] may produce lessened interest in eating. Psychiatric disorders, particularly depression and dementia, have been reported to be the main etiology of UWL in 10–20% of elders [10] and, in nursing home residents, this figure raises to 58% [53]. Depression, which has been more frequently associated with weight loss in older subjects than in younger adults [54], or social isolation, disability/immobility, and low income may cause weight loss due to anorexia and diminished motivation (or feasibility) to buy and cook food [10,12,13]. Drugs and polypharmacy also represent a significant etiologic factor of UWL [1,10–16]. In this study, prescribed and over-the-counter drugs, alone or in combination, were the fundamental cause of UWL in 51 older patients (4% of UWL causes) vs. 3 younger patients. A retrospective study in a US long-term care unit showed that >75% of residents (mean age, 80 years) had been prescribed a drug that likely contributed to weight loss [55]. Among other adverse effects, medications may affect cognition and the subject faculty to eat, or induce xerostomia, anorexia, and nausea [10,56]. Oral problems can only worsen the situation, further contributing to UWL [10,11,15,16,57]. A study of 110 patients (mean age, 77 years) admitted to an US geriatric rehabilitation facility revealed that the number of general oral problems was the strongest predictor of significant UWL within 1 year prior to admission [58]. The prevalence of unexplained UWL in our work was significantly higher in older than younger patients (17 vs. 11%). Despite substantial variations in patients’ age, prospective and retrospective studies have shown that 16–28% of older patients remained without an identifiable cause of UWL after extensive investigations for up to three years [2,4,6,8,10]. Arguably, this could be explained by the cumulative effects of several, commonly interconnected, conditions (eg psychosocial issues, multimorbidity, underlying frailty, oral disorders, polypharmacy, chronic pain) rather than by a serious organic disorder [1,2,4,6–8,10,11,15].

The relevance of unexplained UWL deserves a separate consideration. The issue of unexplained UWL has prompted some amount of research. Based on the reported favorable outcomes of patients with unexplained UWL with initially normal clinical evaluation, it has been argued that a first negative screening with laboratory tests and chest X-ray is reassuring and that serious ‘occult’ disorders, most notably cancer, escaping early detection and uncovered during follow-up are extremely uncommon [1,2,4,8]. However, these claims rely on a few case series with relatively small sample sizes, short or unspecified follow-up periods, and a near total absence of autopsy examinations on dead patients [2–5,8].

A small number of studies have investigated unexplained UWL. In a US prospective study, 1 of 32 (3%) patients was diagnosed with cancer during 1 year of follow-up after an initially negative baseline evaluation [4]. In another study in 306 patients with UWL without specific symptoms (so-called by the authors ‘isolated UWL’), a malignancy was diagnosed in 9 (3%), including one case of pancreatic cancer identified at autopsy, after a follow-up of 1 year [3]. A report in 101 UWL patients disclosed no malignancy among those with unexplained UWL (28%) and a normal baseline evaluation after a follow-up of 9.0 (6.8–16) months [2]. Yet no autopsy was made in any patient who died during the follow-up. Lastly, in the only study to date with the longest follow-up time (mean, 22 [7] months; range, 12–36 months), 26 (16%) of 158 cases were categorized as unexplained UWL [8]. While nonmalignant causes were uncovered in 6 patients during follow-up, the follow-up time was not specified. Also, no deceased patient with unexplained UWL underwent postmortem examination [8]. In the present report, while 375 patients were initially categorized with unexplained UWL, malignancies were detected in 19 of them (5% of cases of unexplained UWL) by extending the initial follow-up period (ie up to 18 months after presentation) an additional 47 months. Overall, all later malignancies were diagnosed between 6 and 28 months after referral, with 9 of 19 being identified more than 12 months after it. Of note, the diagnosis was established postmortem in 14 of the 19 patients. Weight loss in patients with ‘occult’ cancers persisted from presentation in all but 1 case, in which it relapsed after a period of stabilization.

The study has some limitations. First, in addition to the conceivably different aptitudes and attitudes of physicians evaluating UWL, clinical settings, inclusion and definition criteria, and epidemiological and demographic characteristics of evaluable patients may differ substantially. Therefore, the implications of the current study cannot be directly extrapolated to other settings and generalized, which constitutes an inherent limitation. Second, the exclusion of patients lost to follow-up (Fig 1) could potentially introduce bias to the results and subsequent conclusions. While the same might be true for missing data, its minimal extent and nature (ie educational level, monthly income, and Charlson comorbidity index) meant that it was unlikely to affect the results. Third, the long time elapsed in numerous cases between presentation and diagnosis of malignancy was intriguing, mainly considering the lack of relevant abnormalities of repeated tests, predominantly computed tomography and FDG-PET scanning (Table 6). Although 4 patients had some abnormal laboratory tests, these were rather unspecific. The discriminatory utility of laboratory tests for the diagnosis of cancer was found to be modest in a recent prospective study in 290 patients with UWL [19]. However, several reports have concluded that an organic disorder is unlikely when physical examination and baseline tests are normal or irrelevant [2,4,12,13]. It might be reasonable to assume that initially unexplained UWL was causally related to the later malignancies in the minority of patients who had this diagnosis beyond 12 months after referral (Table 6). Yet physiological evidence of a link between UWL and subsequent occurrence of cancer was not available. Likewise, if losses to follow-up and deaths without necropsy are excluded, 95% of patients with unexplained UWL did not have a diagnosis of cancer throughout the full follow-up period (ie 66 months [5.5 years]). Thus, it is possible that some of those who did have such a diagnosis developed it independently of UWL (ie in a posterior stage), in which case there would be no causal link. However, the fact that 18 patients with late malignancies had persistent weight loss since presentation argues against this possibility. Finally, since all apparently ‘occult’ cancers were uncovered (either in necropsy or in living subjects) within 28 months after initial referral (Table 6), our approach of following patients once every 3 months for 5 to 6 years may not have an additional advantage.

Our results have clinical implications. Based on the apparently exceptional occurrence of ‘hidden’ cancer and the favorable prognosis of patients with unexplained UWL who have a negative baseline evaluation [2,4,5,8], it has been recommended a watchful waiting period of 1 to 6 months, while following-up patients for persistent weight loss and new manifestations indicative of an ‘occult‘ disorder [1,2,12–14,59]. Although we agree with these recommendations, which otherwise rely on few and small case series with variable, mostly short, follow-ups, appropriate follow-up periods have not been defined. Moreover, very few necropsies have been done in reported patients with unexplained UWL dying during follow-up [2–4,8]. Based on our findings, ‘occult‘ malignancies detected during a predefined follow-up of up to 66 months after presentation do not seem to be as rare as thought. Nonetheless, the fact that only 1 in 20 patients received such a diagnosis within this period, most notably within the first 28 months after referral for workup, is fairly reassuring. Cancer evading early diagnosis constitute a major concern. In general, the prognosis of malignancies in patients presenting with UWL is very poor with high mortality rates at the short-term because of frequently metastasizing disease when weight loss becomes ostensible [1–3,8,14]. Therefore, despite a normal evaluation and workup and an undisclosed etiology within 1 to 6 months after presentation, evaluation should continue for periods longer than those reported so far through regular follow-up consultations. Furthermore, in order to advance the understanding of unexplained UWL, postmortem studies should be actively pursued in dead patients. In our study, 37 patients who died during follow-up underwent autopsy but 35 did not. Lastly, older patients in whom an obvious cause of UWL is not initially identified should not only be asked about subjective oral difficulties but a careful inspection of the oral cavity should be systematically performed as well as a videofluoroscopy or a swallowing study if the oral examination does not yield conclusive results.

## Conclusion

Among 2677 patients prospectively enrolled over 2 and a half years after referral to a diagnosis clinic of an academic hospital in Barcelona for evaluation of UWL and who underwent a systematic baseline and follow-up approach, 37% were diagnosed with nonmalignant organic disorders, 33% with malignancies, and 16% with psychosocial disorders. Significant differences were observed according to patient age, with oral disorders being the second cause of UWL in patients aged ≥65 years—only after nonhematologic cancers. Of note, while 375 patients were initially diagnosed with unexplained UWL (14% of whole population) because a cause could not be recognized within the first 6 months, ‘hidden’ malignancies were detected in only 19 of them during an extensive follow-up of up to 5.5 years after presentation, with all cases being diagnosed within 28 months after it. Importantly, diagnosis was established at necropsy in 14 cases. Therefore, although unexplained UWL is only rarely a cause for short-term medical alarm, serious underlying disease may be undetectable until death and, consequently, these patients should indeed be followed up regularly (eg annual visits).

In summary, the results in this large cohort of patients with UWL provide new information on the significance of follow-up in the long-term outcome of unexplained cases and on the role of patient age on this clinical entity.
